# Neural interactions in unilateral colliculus and between bilateral colliculi modulate auditory signal processing

**DOI:** 10.3389/fncir.2013.00068

**Published:** 2013-04-19

**Authors:** Hui-Xian Mei, Liang Cheng, Qi-Cai Chen

**Affiliations:** ^1^School of Life Sciences and Hubei Key Lab of Genetic Regulation and Integrative Biology, Central China Normal UniversityWuhan, China; ^2^School of Sport, Hubei UniversityWuhan, China

**Keywords:** inferior collicular neurons, excitatory interaction, inhibitory interaction, bilateral collicular interaction, auditory signal processing

## Abstract

In the auditory pathway, the inferior colliculus (IC) is a major center for temporal and spectral integration of auditory information. There are widespread neural interactions in unilateral (one) IC and between bilateral (two) ICs that could modulate auditory signal processing such as the amplitude and frequency selectivity of IC neurons. These neural interactions are either inhibitory or excitatory, and are mostly mediated by γ-aminobutyric acid (GABA) and glutamate, respectively. However, the majority of interactions are inhibitory while excitatory interactions are in the minority. Such unbalanced properties between excitatory and inhibitory projections have an important role in the formation of unilateral auditory dominance and sound location, and the neural interaction in one IC and between two ICs provide an adjustable and plastic modulation pattern for auditory signal processing.

## INTRODUCTION

In sound reception, auditory signal processing has traditionally been explained by neural interactions of divergent and convergent projections within the ascending auditory system through the interplay between excitation and inhibition (Suga, 1997). Auditory interactions can be found between neurons in one auditory nucleus, bilateral symmetrical auditory structures or nuclei, and even in auditory and non-auditory structures. This implies a neural modulation that plays an important role in maintaining the diversity and accuracy of auditory functions (Mei and Chen, 2010). For example, all sound signals in the range of audible frequency can be perceived by ear, however, we only notice those sounds interested by us and other sound signals that assumed to have no biological significance are filtered by excitatory or inhibitory modulation during transmission upward to different auditory nucleus.

Inferior colliculi (ICs), paired auditory structures, are located between the lower brainstem auditory nuclei and the auditory thalamus in the central auditory pathway. IC receives excitatory and inhibitory inputs from many lower auditory nuclei (Adams, 1979; Adams and Wenthold, 1979; Brunso-Bechtold et al., 1981; Adams and Mugnaini, 1984; Pollak and Casseday, 1989; Covey and Casseday, 1995; Casseday and Covey, 1996), contralateral IC (Malmierca et al., 1995, 2009) and from the primary auditory cortex (AC; Games and Winer, 1988; Herbert et al., 1991; Ojima, 1994; Saldaña et al., 1996; Malmierca and Ryugo, 2011). IC functions as an important relay station, and not only analyzes and integrates sound signals in terms of amplitude, frequency, and time course, etc., from different sources, but also prepares to route these signals to higher level center (Casseday et al., 1994; Jen et al., 1998; Suga et al., 1998; Jen and Zhang, 2000; LeBeau et al., 2001). A number of studies have shown that auditory signal processing and integration in ICs are significantly modulated by the massive descending corticofugal system which adjusts and improves ongoing collicular signal processing in multiple-parametric domains but also reorganizes collicular auditory maps according to the acoustic experience (Jen et al., 1998; Jen and Zhou, 2003; Popelar et al., 2003; Yan et al., 2005; Zhou and Jen, 2007; Ma and Suga, 2008; Suga, 2008; Suga et al., 2010). However, few studies have characterized how neural circuits in or between ICs can affect collicular auditory signal processing and integration. Therefore, in this article, we review recent findings and focus mainly on neural interactions either in one IC or between two ICs.

## EFFECT OF INTERACTIONS BETWEEN NEURONS IN ONE IC IN THE AUDITORY SIGNAL PROCESSING

**FIGURE 1:**
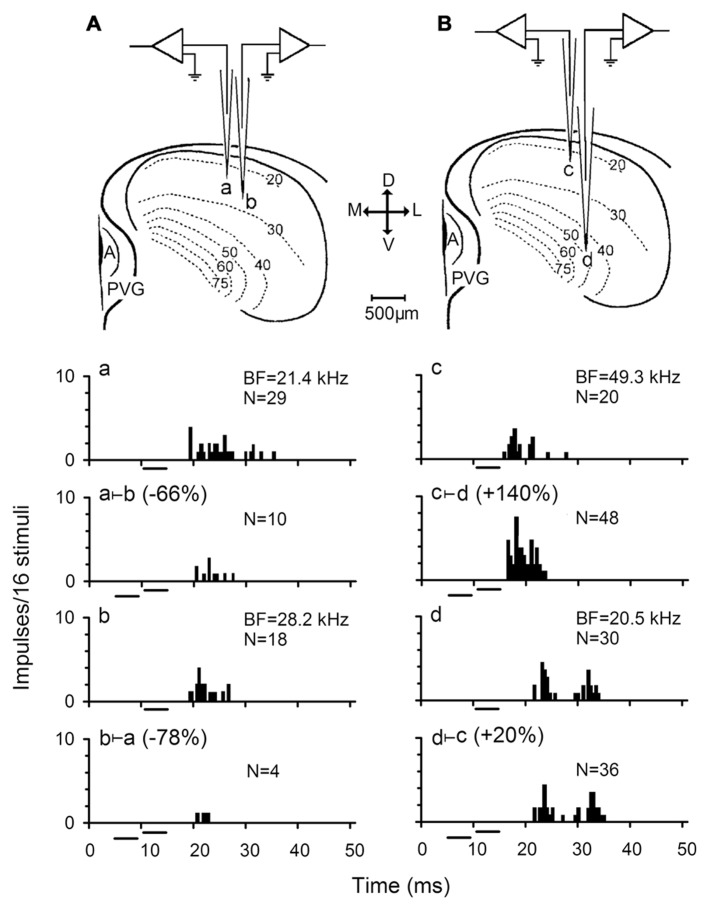
**Responses of two pairs of simultaneously recorded IC neurons.** **(A,B)** Sketches showing the experimental arrangement for simultaneous recording two pairs of IC neurons. a and b represent a pair of IC neurons in iso-frequency laminae while c and d are another pair of IC neurons in non-iso-frequency laminae. M, medial; L, lateral; D, dorsal; V, ventral; A, aqueduct; PVG, paraventricular gray. The firing rates of these neurons in response to a 4 ms best frequency (BF) sound at 10 dB above the minimum threshold (MT) was inhibited (Aa vs Aa ⊢ b, Ab vs Ab ⊢ a) and facilitated (Bc vs Bc ⊢ d, Bd vs Bd ⊢ c) to different degrees when the 10 dB BF sound was preceded by a 4 ms sound at the BF and 20 dB above the MT of its counterpart neuron (abbreviated as the counterpart sound). N: number of impulses. All sound stimuli are shown by short horizontal bars. (based on Jen et al., 2002).

There are extensive intrinsic connections between neurons in one IC such that the IC neurons are likely to be a major source of inputs to other IC cells (Saldaña and Merchán, 1992). Such intercollicular fibers contribute to the formation of the known fibrodendritic laminae in one IC (Herrera et al., 1988, 1989; Oliver and Schneiderman, 1991). How do the neurons inside one auditory center interact with each other? Little is known about this interaction, but immunocytochemical localization demonstrated that one IC contained considerable amounts of glutamic acid, glycine, and glutamate decarboxylase (GAD), an enzyme that catalyzed the decarboxylation of glutamate to γ-aminobutyric acid (GABA), although some of these molecules could have an extrinsic origin (Adams and Wenthold, 1979; Ottersen and Storm-Mathisen, 1984; Vetter and Mugnaini, 1984; Moore and Moore, 1987; Roberts and Ribak, 1987; Caspary et al., 1990; Merchán et al., 2005). The presence of these excitatory and inhibitory transmitters suggests extensive interactions and modulations between neurons in one IC, because excitation and inhibition are the two most important neural interactions that modulate auditory signal processing by increasing and decreasing responses of auditory neurons. To study the effect of neural interactions on sound amplitude and frequency selectivities of IC neurons, the auditory responses including the rate-intensity function (RIF) and frequency tuning curve (FTC) of each IC neuron in two simultaneously recorded IC neurons (or paired neurons) were examined under two-tone stimulation conditions. A pair of electrodes was used to simultaneously record two IC neurons in the same iso-frequency lamina or different iso-frequency (non-iso-frequency) laminae of the IC (**Figure 1**). A modulating tone with the best frequency (BF) of one of the paired IC neurons was delivered prior to a probe tone. This two-tone stimulating paradigm provided an opportunity to examine how a neuron activated by its BF sound might affect the response of the other neuron in amplitude and frequency domains. In particular, this procedure allows us to study the possible correlation of each pair of neurons in signal processing. For example, when a pair of IC neurons was stimulated by their two BF tones, the response of one IC neuron was either inhibited (two-tone suppression, **Figure 1A**) or facilitated (two-tone facilitation, **Figure 1B**) by the other. It has been reported that the proportion of neurons inhibited by interactions between simultaneously recorded neurons was always higher than that of facilitated neurons (Jen et al., 2002; Wu and Jen, 2008). Thus, the high level of inhibition in IC is basically similar to that in other reports (Vater et al., 1992; Suga, 1995; Fuzessery and Hall, 1996; Zhou and Jen, 2000; Lu and Jen, 2002; Mayko et al., 2012).

Further testing of inhibitory interactions on responses of the paired neurons revealed that the percent two-tone suppression of auditory responses decreased significantly with BF and recording depth differences between paired IC neurons (Jen et al., 2002). This observation is similar to a study in which auditory spatial selectivity of IC neurons was studied under two-tone stimulation conditions (Zhou and Jen, 2000). It was proposed that this phenomenon might be caused by the tonotopic organization of IC neurons, and that inputs from neurons with small BF differences arrive earlier with less attenuation than neurons with large BF differences (Jen et al., 2002). On the other hand, this observation also suggests a gradient of decreasing two-tone suppression along the dorsoventral axis of the IC (Schreiner and Langner, 1997). However, the neural basis underlying this observation remains to be explored.

Because the two-tone stimulation was based on the BFs of two simultaneously recorded neurons, two-tone suppression and facilitation might be thought to be caused by interactions between the two simultaneously recorded neurons activated by their respective BF sounds. Since IC neurons are tonotopically organized, interactions between the IC neurons are actually interactions between frequency laminae or bands. For a pair of IC neurons simultaneously recorded in big brown bat, a sound with the BF of one neuron could modulate the frequency tuning of another neuron by sharpening or broadening it’s FTC (Wu et al., 2004). The pairs of neurons involved in frequency band interaction are not only within the same frequency band, but also across different frequency bands. The sharpening degrees of neurons within the same frequency band are higher than those of neurons across frequency bands. It was also found that the strength of frequency band interactions was weaker near the BF but gradually increased with frequency away from the BF of FTC (Wu et al., 2004). Moreover, FTCs of neurons with a BF of 20–30 kHz are most strongly sharpened which is similar to that observed in the chinchilla (Biebel and Langner, 2002).

These data suggest that IC neurons are highly correlated during frequency analysis such that frequency selectivity of the IC neurons is improved through inhibition while the spectrum of frequency sensitivity of other IC neurons is enhanced through excitation.

**FIGURE 2:**
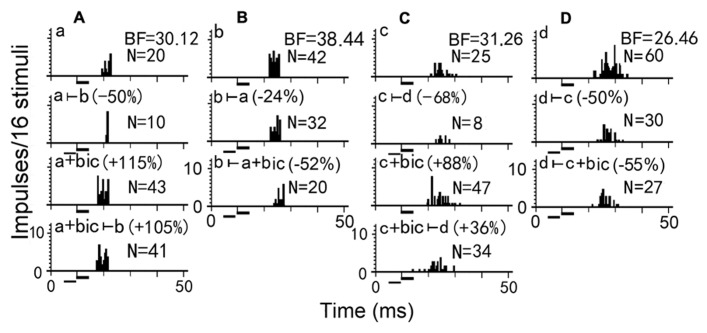
**Two-tone suppression on responses of two pairs of simultaneously recorded IC neurons before and during bicuculline (bic) application.** Presentation of a counterpart sound decreased the firing rates of each IC neuron in the **(A,B)** pair or **(C,D)** pair (Aa, Bb, Cc, Dd vs Aa ⊢ b, Bb ⊢ a, Cc ⊢ d, Dd ⊢ c). When bicuculline was applied to neurons A and C, the number of impulses increased (Aa vs Aa+bic, Cc vs Cc+bic). The presentation of a counterpart sound during bicuculline application to neurons A and C decreased the number of impulses only slightly in neuron A but substantially in neuron C (Aa+bic vs Aa+bic ⊢ b, Cc+bic vs Cc+bic ⊢ d), however, decreased the number of impulses substantially in neuron B and slightly in neuron D (Bb vs Aa+bic ⊢ b, Cc+bic vs Cc+bic ⊢ d). N: number of impulses. All sound stimuli are shown by short horizontal bars. (Wu and Jen, 2008)

To further explore the mechanism underlying the effect of two-tone suppression on the responses of two simultaneously recorded neurons, bicuculline (an antagonist of GABA_A_ receptor) was applied to one of the paired IC neurons in big brown bat to abolish GABAergic inhibition (**Figure 2**). Using a pair of neurons (A and B, for example), when bicuculline was applied to neuron A, it’s number of impulses was greatly increased (**Figure 2** Aa vs Aa+bic), and the two-tone suppression was completely removed in neuron A (**Figure 2** Aa+bic vs Aa+bic ⊢ b), but was stronger in neuron B (**Figure 2** Bb ⊢ a vs Bb ⊢ a+bic). Thus, the degree of response inhibition decreased in the bicuculline-applied neuron but increased in the paired neuron, suggesting that GABAergic inhibition directly mediated the inhibitory interactions between two simultaneously recorded or paired IC neurons (Wu and Jen, 2008, 2009). However, for another pair of neurons C and D, the number of impulses greatly increased following bicuculline administration to neuron C (**Figure 2** Cc vs Cc+bic), but the two-tone suppression was only partly abolished in neuron C (**Figure 2** Cc+bic vs Cc+bic⊢ d), and was slightly increased in neuron D (**Figure 2** Dd⊢ a vs Dd⊢ c+bic). A previous study in big brown bat indicated that IC neurons with GABA_A_ receptors are mostly distributed in the dorsomedial region but are sparsely distributed in the ventrolateral region which is mostly distributed with neurons containing glycine receptors (Fubara et al., 1996). Therefore, the degree of GABA-mediated two-tone suppression would progressively decrease along the dorsoventral axis of the IC. In brief, when an IC neuron is excited, it may inhibit other neighboring neurons to stand out as the best in the neurons through inhibitory interaction. These inhibitory interactions between neurons in one IC improves auditory sensitivity during auditory signal processing.

## BILATERAL COLLICULAR INTERACTION IN AUDITORY SIGNAL PROCESSING

**FIGURE 3:**
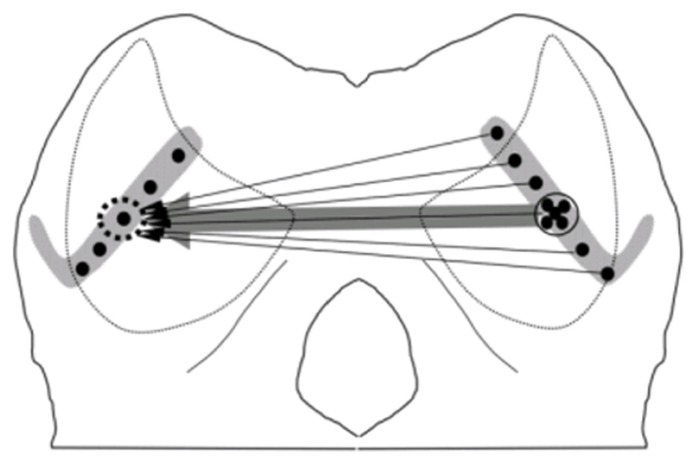
**Schematic wiring diagrams of the commissural connections.** In the central nucleus of IC (ICc), the retrograde labeling of neurons demonstrated that an injection into one point on the lamina (dotted circle, left IC) retrogradely labeled neurons over the whole extent of the contralateral lamina, consistent with a divergent pattern of connections (thin arrows). The density of the projection is centered on a point matching the position of the tracer injection which is consistent with a point-to-point-weighted wiring pattern (thick arrow; Malmierca et al., 2009).

Many previous studies have clearly shown the anatomical connections between two ICs through the commissure of IC (CoIC). Injecting retrograde tracer in one IC demonstrated that commissure neurons in the central nucleus of IC (ICc) sent projections or fibers to the central nucleus, dorsal and lateral cortices of opposite IC. The commissural fibers ending in the contralateral IC to the injection point formed a laminar plexus that was symmetrical to the ipsilateral plexus, and interconnected mirror symmetric regions of the ICs representing similar frequency bands (Saldaña and Merchán, 1992; Malmierca et al., 1995). Even in the ICc, retrograde labeling of neurons demonstrated that commissural neurons send a divergent projection to the whole extent of the contralateral lamina, which resulted in a V-shaped axonal plexus that covered most of the ICc laminae and extended into the dorsal and lateral cortices. However, the density of the labeled commissurally projecting neurons was weighted toward a point that matched the position of the corresponding tracer injection into the contralateral IC, which is consistent with a point-to-point pattern (**Figure 3**). The coexistence of point-to-point and divergent projections suggest that CoIC is likely to be involved in interactions between specific regions of corresponding frequency band laminae as well as in integration across the laminae. (Malmierca et al., 2009).

An immunocytochemistry study in CoIC (Saint Marie, 1996) demonstrated the presence of both excitatory projections mediated by glutamate and inhibitory projections mediated by GABA. Injections of D-[3H] aspartate which is considered a selective marker for glutamatergic synapses, suggested that some glutamatergic endings in the IC originated from the opposite IC in the chinchilla. Studies that combined tract-tracing with horseradish peroxidase (HRP) and immunocytochemical labeling for GABA, found that double labeled neurons were mostly in the contralateral IC following a tracer injection into the ipsilateral IC in rat. These GABAergic CoIC could exert a direct monosynaptic inhibitory influence on their contralateral counterparts (González-Hernández et al., 1996; Hernández et al., 2006).

These anatomical findings are consistent with an electrophysiological study that concentrated on the interactions between two ICs. *In vitro* whole cell recording of IC neurons demonstrated that an excitatory and inhibitory postsynaptic current (EPSC and IPSC) was evoked by direct stimulation of the CoIC. The addition of GABAergic or glycinergic antagonists to CoIC could reduce the IPSC to various degrees, even there was a strong inhibitory input that was almost exclusively GABAergic. Furthermore, ionotropic glutamic receptor antagonists reduced both the EPSC and IPSC. This indicated that much of the inhibitory input appears to be mediated by interneuronal connection (Moore et al., 1998). Inactivation of excitatory CoIC could inhibit recorded IC neurons by direct elimination of the excitation and facilitate recorded neurons by disinhibiting inhibitory synapse of interneurons.

Bilateral collicular interaction between two ICs in auditory signal processing were examined using extracellular recordings *in vivo*. Malmierca et al. (2003, 2005) blocked the transmission of excitatory fibers in CoIC by means of local hydraulic injection of kynurenic acid (KA; a non-specific glutamatergic receptor antagonist) into one IC and observed changes in the frequency response area, number of impulses and monotonicity of neurons located in the corresponding region of the contralateral IC. These studies indicated bilateral collicular interactions in the corresponding frequency laminae between the two ICs that were mediated by CoIC. Consistent with the result of whole cell recording, focal injection of KA in one IC both decreased and increased the number of impulses in the opposite IC neurons. This provided further evidence for an inhibitory influence mediated by inhibitory interneuronal connection.

Moreover, our recent study also demonstrated that focal electrical stimulation of one IC produced widespread inhibition and focused excitation of responses in contralateral IC neurons. The excitatory modulation of bilateral collicular interactions expands the RIFs and FTCs of facilitated IC neurons but decreased the slope of their RIFs and Q_10_ value of their FTCs for wider amplitude and frequency responses to sound stimuli. Conversely, the inhibitory modulation of bilateral collicular interaction sharpens the RIFs and FTCs of inhibited IC neurons but increased the slope of their RIFs and Q_10_ value of their FTCs for sharper sensitivity to sound amplitude and frequency (Mei et al., 2012b; Cheng et al., 2013). It is also suggested that the small proportion of bilateral collicular excitatory interactions between neurons in corresponding frequency laminae and the large proportion of bilateral collicular inhibitory interactions between neurons in different frequency laminae may be involved in the formation of binaural neurons (i.e., excitation–excitation, EE neurons that can be excited by same BF sound stimulation to either ear; excitation–inhibition, EI neurons that are strongly excited by sound stimulation to the contralateral ear and are inhibited by sound stimulation to the ipsilateral ear; Mei et al., 2012a). The possible neural pathway may be described that the excitation from ipsilateral ear can cross to the contralateral IC in a lower auditory nucleus and then to the ipsilateral IC via facilitatory or inhibitory CoIC, respectively. The unbalanced properties between excitatory and inhibitory projections have a very important role in the formation of unilateral auditory dominance and sound location.

**FIGURE 4:**
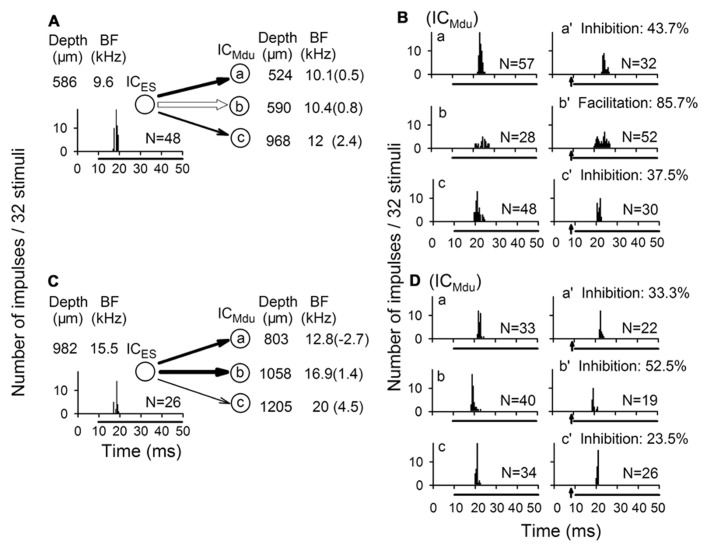
**Modulation of response of IC neuron (IC_**Mdu**_) during focal electrical stimulation of the contralateral IC neuron (IC_**ES**_). **(A,B)**** Focal electrical stimulation of one IC_ES_ neuron produced inhibition of two IC_Mdu_ neurons but produced facilitation of another IC_Mdu_ neuron. **(C,D)** Focal electrical stimulation of another IC_ES_ neuron produced inhibition of all three IC_Mdu_ neurons studied. The BF of all six IC_Mdu_ neurons progressively increased with recording depth, and the percent inhibition was closely correlated with BF difference. N, number of impulses; %, percent inhibition or facilitation; of response of each IC_Mdu_ neuron. Arrow: focal electric stimulation (Mei et al., 2012b).

In accordance with the anatomical data of point-to-point and divergent projections between two ICs, focal electrical stimulation of one neuron modulated the responses of three contralateral neurons (**Figures 4A** vs **4B** and **4C** vs **4D**). Each of three contralateral IC neurons was sequentially isolated at a different depth with a progressive increase in BF. The degree of bilateral collicular interaction was dependent upon the BF difference between the electrically stimulated IC neurons and the modulated IC neurons. The percent modulation in the number of impulses was larger for the neuron with a smaller BF difference than for the neuron with a larger BF difference (Mei et al., 2012b).

In addition, after the focal electrical stimulation was delivered for 30 min, a long term shift in an IC neuron’s BF was induced which remained for as long as 150 min and decreased with time (Cheng et al., 2013). Therefore the bilateral collicular interaction modulates both auditory signal processing and auditory plasticity of IC neurons that is similar to the corticofugal modulation of IC neurons (Jen et al., 1998; Ma and Suga, 2001; Suga et al., 2002; Yan and Ehret, 2002; Jen and Zhou, 2003; Yan et al., 2005; Zhou and Jen, 2007). Since the BF-dependent modulation of bilateral collicular interaction is not entirely comparable to the egocentric selectivity of corticofugal modulation, further studies are required to determine whether the modulation effect of bilateral collicular interactions might also be mediated through corticofugal feedback loops.

Interestingly, following reciprocal electrical stimulation of pairs of neurons, respectively, in two ICs, we found that the bilateral collicular interaction was either reciprocal or unilateral. However, after HRP deposits were made in CoIC, regions of the IC supplying fibers to the commissure were not the main targets for the terminals of these fibers, which suggested that interconnections of the ICs through their commissure were complementary, rather than reciprocal (Aitkin and Phillips, 1984).

## PROSPECTS

Neural interactions are of great interest because of their contribution to sensory information processing, neural functional integration and neural modulation. As for the auditory midbrain, neural interactions were found both in one IC and between two ICs, even in unilateral iso-frequency and non-iso-frequency laminae as well as bilateral corresponding and non-corresponding frequency laminae. Generally, there is a large percentage of inhibitory interactions but a small percentage of excitatory interactions, which is likely because of the presence of many inhibitory interneurons. These excitatory and inhibitory interactions in or between ICs modulate auditory signal processing in amplitude and frequency domains, and provide an adjustable and plastic modulation pattern for the auditory signal processing of ICs. However, many details, such as neural plasticity of the structure and function as well as cellular and synaptic mechanisms of the neural modulation underlying neural interactions in auditory signal processing, remain unclear and require further study. We have sufficient reasons to believe that new knowledge about the various neural interactions will be obtained with successive studies. Thus, the studies of neural interactions in one IC and between two ICs are in the ascendancy.

## Conflict of Interest Statement

The authors declare that the research was conducted in the absence of any commercial or financial relationships that could be construed as a potential conflict of interest.
